# Complex gastroschisis with apple peel jejunoileal atresia, primary closure, and Santulli procedure as a surgical alternative. Case report

**DOI:** 10.1016/j.ijscr.2022.107095

**Published:** 2022-04-19

**Authors:** José Luis Castillo - Clavijo, Patricio F Gálvez - Salazar, Mariana Ángel-Correa, Valentina Montañez-Azcárate, Diego Alfredo Palta - Uribe, Luis Mauricio Figueroa - Gutiérrez

**Affiliations:** a Auxiliary Professor, Hospital Universitario del Valle-Evaristo García, Universidad del Valle, Cali, Colombia; b PGY-1, Pediatric Surgery Fellowship Program, Universidad del Valle, Cali, Colombia; c School of Medicine, Universidad del Valle, Cali, Colombia; d Chief of Pediatric Surgery Hospital Universitario del Valle, Auxiliary Professor Universidad del Valle, Cali, Colombia; e Assistant Professor, Universidad del Valle, Cali, Colombia

**Keywords:** Apple peel intestinal atresia, Intestinal atresia, Gastroschisis, Pediatric surgery, Neonatal surgery, Case report

## Abstract

**Background:**

Gastroschisis is a closure defect of the abdominal wall classified as complex when it presents with necrosis, volvulus, or atresia of the gastrointestinal tract. Jejunoileal atresia is caused by abnormal closure, discontinuity, or narrowing of the intestine. Apple Peel or type IIIb is the rarest presentation, with an incidence of 1.3 per 10,000 live births. In addition to presenting a high mortality rate.

**Presentation of case:**

We present a preterm newborn patient of 30 weeks with a diagnosis of gastroschisis and jejunoileal atresia type IIIB. The congenital wall defect was closed in the first surgical stage, and he was then taken at four weeks to correct the atresia. In the second surgery, we found a difference in intestinal calibers of 8:1, and the surgical team decides to perform remodeling of the proximal sac with a mechanical stapler and perform anastomosis using the Santulli technique.

On day 6 of life, enteral feeding began through a nutrition tube localized under intestinal anastomosis with progressive nutritional increase.

Subsequently, intermittent and progressive occlusion of the stoma was performed, leading the patient to a definitive surgical closure one month later.

**Conclusions:**

The mortality rate for gastroschisis and complex intestinal atresia is high. Advances in prenatal diagnosis, neonatal intensive care, and proper surgical correction are crucial to improving survival rates. The Santulli procedure is a surgical alternative for intestinal atresias with a caliber discrepancy greater than 4 to 1 or when the characteristics of the distal part do not allow a primary anastomosis to be performed.

## Highlights

• Gastroschisis associated with volvulus, necrosis, or any atresia of the gastrointestinal tract is classified as complex. It must have an optimal multidisciplinary and surgical treatment to obtain lower morbidity and mortality.
• The Santulli procedure is a surgical technique that can be used as a therapeutic option for complex intestinal atresia when the ratio of the intestinal ends is greater than 4:1 since it allows early enteral nutrition and early and progressive dilation of the intestinal distal end.
• We must not forget that the perioperative and surgical treatment of complex gastroschisis and intestinal atresia must be individualized for each patient, depending on their clinical situation.

## 1 Introduction and importance

This work has been reported according to the SCARE criteria [1].

Jejunoileal apple peel atresia is the rarest presentation of small bowel atresias, with a reported incidence of 1.3 per 10,000 live births [2]. It is produced by a vascular disruption that induces intestinal ischemia and necrosis, forming two proximal and distal blind sacs adhered to the mesentery. Associated risk factors are smoking mothers, psychoactive substances, Hirschsprung's disease, and abdominal wall defects. Gastroschisis associated with intestinal atresia is a complex pathology characterized by high mortality, so treatment must be individualized [3].

The mortality rate is the highest for small bowel atresias, especially in developing countries, due to poor access to prenatal diagnosis and perioperative medical and surgical support. These are essential to improve the survival rate [4], [5]. Therefore, type IIIB jejunoileal atresia should be considered a life-threatening malformation that requires multidisciplinary treatment.

Knowledge about the perioperative approach, surgical techniques, support measures in the neonatal intensive care unit, and complications associated with the stoma are fundamental in treating this pathology.

Reporting the successful management of complex gastroschisis through primary closure and an IIIB intestinal atresia with a difference in intestinal calibers greater than 8:1 through the Santulli technique allows us to show a therapeutic alternative in these patients.

## 2 Case report

We present a preterm newborn male patient of 30.5 weeks of gestation and weight of 1775 g, the son of an adolescent mother with a history of exposure to tobacco.

At 26.6 weeks of gestation during prenatal care, obstetric ultrasound showed signs of gastroschisis associated with intestinal atresia, finding intestinal loops in the amniotic cavity with a documented dilation of 17 mm. The mother continued periodic ultrasound control by the perinatology team, showing progressive intestinal dilatation.

**Fig. 1:**
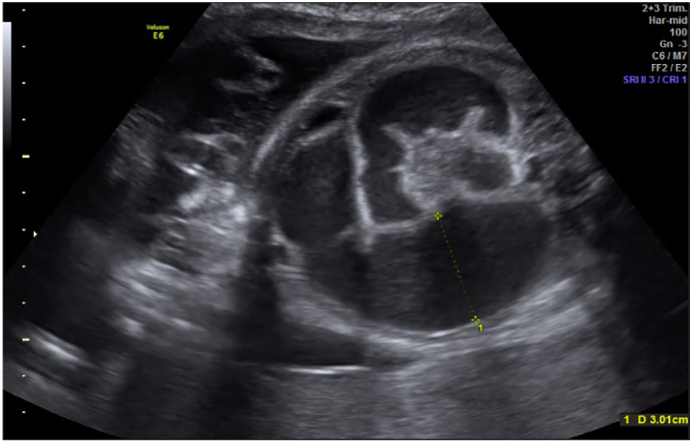
Intestinal atresia identified by prenatal ultrasonography, showing dilation of intestinal loops of 30 mm and interloop edema.

During the last prenatal control, the patient presented fetal distress. The ultrasound shows signs of intestinal distress and dilation of 30 mm (Fig. 1). Due to this, the pregnancy was terminated by cesarean section.

**Fig. 2:**
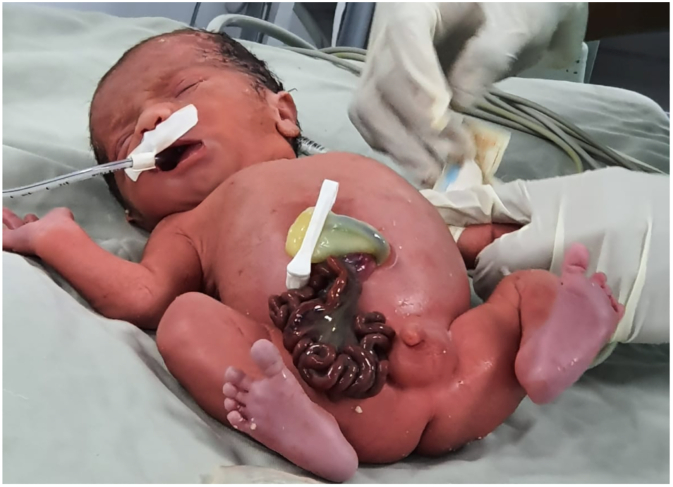
Preterm newborn with gastroschisis and intestinal atresia.

The newborn was taken to the operating room to correct gastroschisis by primary closure under general anesthesia. During surgery, jejunoileal atresia is evident. The pediatric surgery team decided to defer the correction of the jejunoileal atresia due to the patient's prematurity, low birth weight, and inflammation of the intestinal loops, which increase the risk of complications (Fig. 2).

The patient was admitted to the neonatal intensive care unit for postoperative follow-up and total parenteral nutritional support. During hospitalization, the patient did not present complications associated with nutritional support. Abdominal X-ray showed intestinal loop distention with air-fluid levels, compatible with jejunal atresia. Upon reaching 34 days of age and 2670 g, we decided to perform surgical correction of the jejunoileal atresia.

**Fig. 3:**
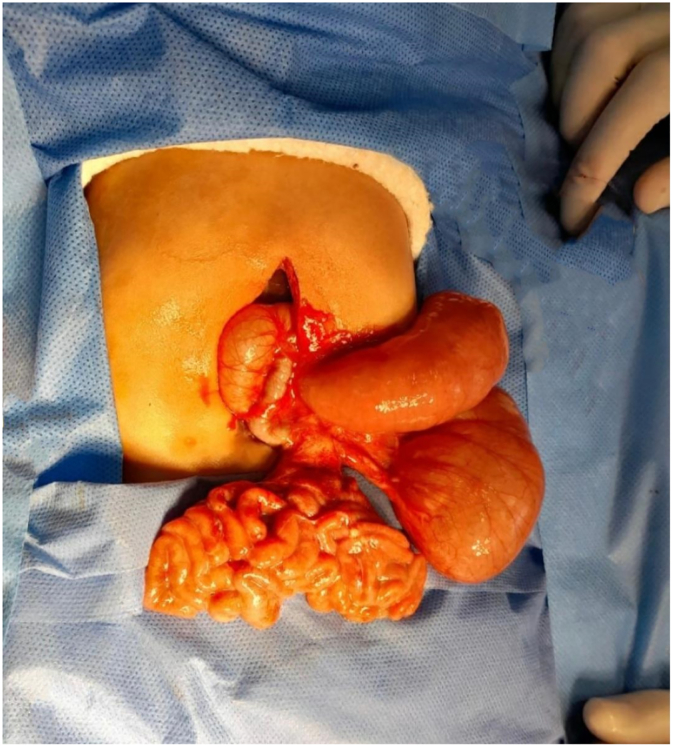
Intraoperative findings of type IIIB jejunal atresia. The defect is observed in “Apple peel” or “Christmas tree.”

During surgery, the patient presented fixed intra-abdominal adhesions of the intestine to the abdominal wall, jejunal atresia measuring 50 cm from the angle of Treitz with a proximal-distal ratio of 8:1, the distal segment of the small intestine measuring 70 cm in length; fulfilling criteria for type IIIB intestinal atresia (Fig. 3).

**Fig. 4:**
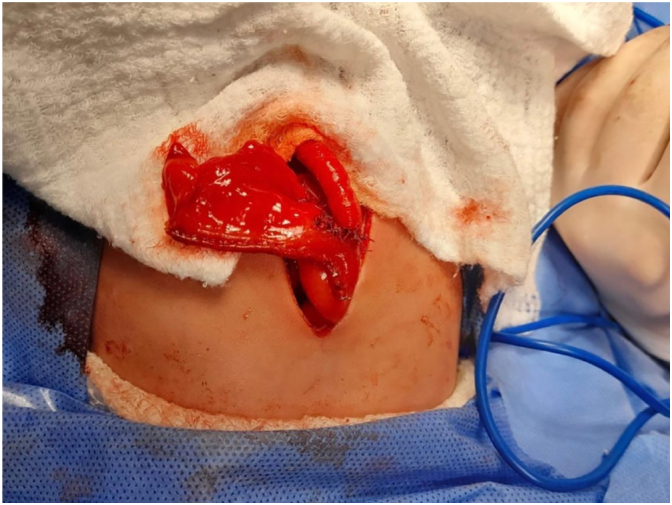
Lateral-to-terminal jejuno-jejunal anastomosis.

We performed the correction of the intestinal atresia using the technique described by Santulli due to the proximal-distal ratio and the intestinal length of the distal segment. The atretic segment was resected, and the proximal sac was remodeled by narrowing with a 60-mm mechanical suture and lateral-to-terminal jejune-jejunal anastomosis (Fig. 4). In addition, a proximal jejunal segment stoma is performed to ensure adequate decompression, and a feeding tube is inserted into the jejunum below the intestinal anastomosis.

The patient had an adequate postoperative evolution, enteral stimulation by feeding tube was started six days after surgery, and the output per stoma during the first week was more than 1 ml/kg/h. However, the patient did not present dehydration or electrolyte disturbances. Subsequently, the jejunostomy was intermittently occluded to achieve progressive dilation of the distal end. Enteral feeding was started, reaching the nutritional goals.

One month after the correction of the intestinal atresia, we closed the stoma in the operating room under general anesthesia without surgical complications. The patient was discharged and is currently undergoing regular outpatient follow-up. The patient is six months old, without presenting intestinal obstruction, and with adequate weight and height for his age.

## 3 Clinical discussion

Gastroschisis is a malformation of the abdominal wall that occurs in isolation, and with a mortality of 10%, the treatment is surgical, either by primary closure or by stages [3]. When associated with intestinal atresias, it is considered complex gastroschisis due to the number of surgical interventions and the increased morbidity and mortality of these patients. Therefore, the surgical treatment for each patient must be individualized to obtain an optimal result with the least possible complications.

Small bowel atresia is a gastrointestinal defect characterized by abnormal closure, discontinuity, or narrowing of the duodenum, jejunum, or ileus [6]. Within the small bowel atresias, there is jejunoileal atresia, the most common form of intestinal obstruction in the neonatal and infant age group, and has an incidence of 1: 300 to 1:1500 [4], [5].

Intestinal atresia can be caused by abnormal rotation, volvulus, invaginations, hernias, and segmental vascular flow interruptions [3]. 30% to 70% of newborns with jejunoileal atresia are born prematurely and are associated with chromosomal abnormalities in less than <1% [4], [7].

Prenatal diagnosis of intestinal atresia is made by ultrasound. One of the first manifestations of this anomaly is polyhydramnios during pregnancy, but it is not specific [3]. In addition, prenatal ultrasound can show findings of distended and fluid-filled intestinal loops, both highly suggestive of this pathology [7]. Between 30 and 60% of patients with intestinal atresia have a prenatal diagnosis [4], [7].

According to Louw, jejunoileal atresias are classified into four groups depending on their anatomical characteristics [7], [8]. Type III is subdivided into IIIa and IIIb, according to Grosfeld et al. [7], [8]. Jejunoileal atresias type IIIb, IIIa, and IV are considered complex [4].

Type IIIb or also known as “Apple peel” or “Christmas tree,” is the rarest of the presentations, providing 7–10% of the atresias of the small bowel [4]. Being more frequent in women, with a ratio of 1.6:1 [4]. In Latin America, it has an incidence of 1.3 per 10,000 live births [2]. Apple peel atresia describes atresia just below the duodenojejunal flexure, with the remaining small intestine wrapped around the ileocolic artery [7]. The superior mesenteric artery is absent beyond the middle colic vessels [7].

The mortality of neonates with this type of atresia is higher than the others [5]. The prognosis is generally related to the total length of the remaining intestine and the presence of an intact ileocecal valve [7]. Type III atresia is frequently associated with significant loss of intestinal length and short bowel syndrome in up to 74% of patients with apple peel atresia [4], [7].

Jejunoileal atresia IIIB is more frequently associated with other malformations or anomalies; there may be intestinal malrotation in 50% of these cases [4]. Failure to search for and diagnose other associated abnormalities may also contribute to mortality in patients with jejunoileal atresia [9].

Volvulus, internal hernias, strictures, intussusception, intestinal atresias, meconium peritonitis, microcephaly, leukoma corneal atresia, polysplenia, limb defects, situs inversus, and immunodeficiencies are more frequent in apple skin atresia, producing more secondary complications [4].

There are various surgical techniques for treating jejunoileal atresias, such as resection and end-to-back anastomosis in patients with a remnant intestine of relatively average length. In simple atresias, intestinal resection and primary anastomosis with or without enteroplasty are gold standard surgical techniques [10].

In contrast, complex atresias remain a challenge due to the risk of creating poorly functional anastomosis that can lead to significant morbidity and mortality for trying to preserve a longer bowel length. Decompression and functional stoma can be used. Different surgical options described in the literature are the Santulli and Bishop Koop techniques and a T-tube enterostomy, with variable achievement rates and possible complications [10].

First described in 1961 for the treatment of intestinal atresia, the Santulli procedure has been proposed in cases of necrotizing enterocolitis in very low weight patients up to 30 weeks of gestation and weighing up to 1095 g with good results [11], [12]. This procedure can be used in different clinical conditions ranging from intestinal atresia to midgut volvulus and intestinal perforation [13]. The Santulli technique is performed when the discrepancy between the intestinal segments is greater than 4 to 1 or when the appearance or trophism of the distal part is not satisfactory for performing a primary anastomosis.

**Fig. 5:**
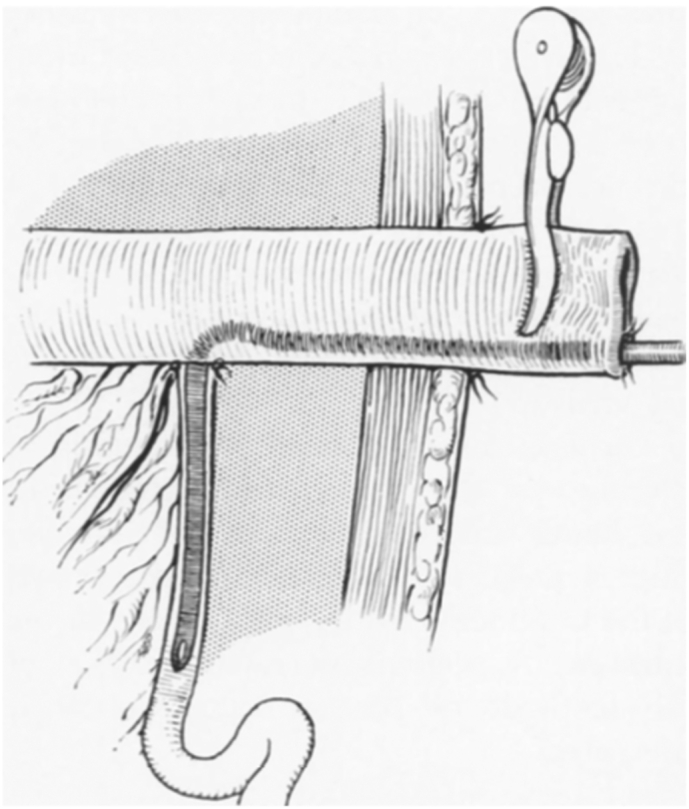
“End-to-side anastomosis with proximal end type of enterostomy and catheter in distal bowel.”

The Santulli technique remodels the proximal bowel segment, anastomosed lateral-to-terminal with the distal bowel segment. Additionally, an enterostomy is performed with the proximal part (Fig. 5) [11]. Its advantages include the rapid use of the proximal intestine with trophic enteral feeding and the decompression of the proximal part, allowing the growth of the distal portion [14]. Additionally, it promotes intestinal growth and maturation, restores enterohepatic circulation, and preserves the intestinal microbiota, reducing the risk of cholestasis, sodium depletion, and metabolic acidosis.

Taken from: SANTULLI T V, BLANC WA. Congenital atresia of the intestine: pathogenesis and treatment. Ann Surg [Internet]. 1961 Dec; 154 (11): 939–48. Available from: http://www.ncbi.nlm.nih.gov/pubmed/14497096.

The second surgery is the closure of the stoma. It is performed when the patient has adequate enteral nutrition, growth of the small intestine, and intestinal production of fewer than 0.8 ml/kg/day or 20 ml/kg/day [15], [16].

Regarding mortality, the survival rates of jejunoileal atresias have improved considerably. In 1950 mortality was close to 90%, while in the last two decades, reports from developed countries show survival rates of 95% or more [4], [5]. Advances have influenced this increased survival in anesthesia, neonatal intensive care, and the availability of total parenteral nutrition [5]. Developing countries that do not have the same availability of these resources still have high mortality rates [5].

Apple peel atresia has higher mortality than other atresias, and it is considered a life-threatening malformation since it is always associated with substantial morbidity and mortality [4]. In developing countries, most cases of the mortality reports are related to sepsis, either caused by anastomotic leaks or related to total parenteral nutrition [4].

## 4 Conclusions

The management of the patient with gastroschisis associated with life-threatening intestinal atresias such as type IIIb and IV is a challenge for pediatric surgeons; this is associated with high morbidity and mortality. Therefore, patients with this pathology require multidisciplinary and individualized treatment.

The Santulli procedure consists of two phases: the first to correct the atresia and create a stoma, and the second to close the stoma. Although it is not the gold standard of treatment, it may be a therapeutic option in patients with a difference in intestinal caliber greater than 4:1.

The Santulli procedure requires strict postoperative monitoring due to the risk of dehydration and electrolyte imbalance. Closure of the stoma is recommended when the patient has oral feeding, adequate weight gain, and stoma production (<20 ml/kg/d or 0.83 ml/kg).

## Funding

Have no funding to report.

## Ethical approval

The authors declare that we obtained permission from the ethics committee in our institution.

## Consent

Written informed consent was obtained from the patient to publish this case report and accompanying images. A copy of the written consent is available for review by the Editor-in-Chief of this journal on request.

## Author contribution

José Luis Castillo: Data curation.

Patricio Gálvez Salazar: Conceptualization, Data curation, Formal analysis.

Mariana Ángel-Correa: Conceptualization, Data curation, Formal analysis.Valentina Montañez-Azcárate: Conceptualization, Data curation, Formal analysis, References.

Diego Alfredo Palta Uribe: Data curation, Formal analysis.

Luis Mauricio Figueroa: Conceptualization, Data curation, Formal analysis.

## Registration of research studies

The authors declare that the patient consented to publish this case, and as this is a case report, no human participants were involved in a study.

## Guarantor

Luis M Figueroa G Assistant Professor Hospital Universitario del Valle-Evaristo García, Universidad del Valle, Cali, Colombia.

## Declaration of competing interest

The authors declare that there is no conflict of interest regarding the publication of this article.
